# Psychopathic Traits in Adult versus Adolescent Males: Measurement Invariance across the PCL-R and PCL:YV

**DOI:** 10.3390/bs14080672

**Published:** 2024-08-02

**Authors:** Darlene A. Ngo, Craig S. Neumann, J. Michael Maurer, Carla Harenski, Kent A. Kiehl

**Affiliations:** 1 Department of Psychology, University of North Texas, 1155 Union Circle #311280, Denton, TX 76203, USA; darlenengo@my.unt.edu; 2 Mind Research Network, 1101 Yale Blvd. NE, Albuquerque, NM 87106, USA; mmaurer@mrn.org (J.M.M.); charenski@mrn.org (C.H.); kkiehl@mrn.org (K.A.K.)

**Keywords:** psychopathy, development, measurement invariance, PCL-R, PCL:YV, exploratory structural equation modeling

## Abstract

Both the Psychopathy Checklist–Revised (PCL-R) and the Psychopathy Checklist: Youth Version (PCL:YV), respectively, are established instruments for assessment of psychopathy and development of psychopathic propensity. To reliably compare scores from both instruments, measurement invariance must be established. The current study involved a combined sample of 1091 male participants (adults = 813; adolescents = 278) from correctional facilities in New Mexico. An exploratory structural equation modeling (ESEM) framework was used to test for measurement invariance. The four-factor ESEM model demonstrated good fit for the combined and individual samples. Results from the multiple group ESEM provide evidence for generally strong invariance, with equivalent factor loadings and thresholds. Adolescents exhibited decreased latent interpersonal traits but increased latent features on other PCL factors (affective, lifestyle, and antisocial) compared to adults. Findings suggest that the four-factor model and the measurement of psychopathic traits remain consistent across age groups. Implications of the findings within research and clinical contexts are discussed.

## 1. Introduction

Psychopathy is a chronic disturbance in personality that is comprised of manipulative, deceptive, callous, impulsive, and antisocial tendencies [1]. The prevalence of psychopathic traits is estimated to be between 1% and 3% in the community [2,3] and up to 25% in forensic settings [4]. Psychopathy is associated with negative correlates and outcomes such as emotion dysregulation [5], violence, risk for criminality [6,7,8], and comorbid substance dependencies [9]. Because psychopathic features emerge early and the pathology is thought to be lifelong [10,11] studying the development of psychopathy throughout the lifespan is an integral focus in research.

Considerable research on the development of psychopathy focused on callous-unemotional (CU) traits in children and youth, and until recently, less attention was devoted to other psychopathy-related traits [2,12]. The intense focus on CU traits is due in part to the perspective that disturbances in empathy are proposed to be a primary basis for development of psychopathic personality [13]. However, questions about the specificity of the empathy–psychopathy link [12] and the veracity of empathy assessments were raised [14,15,16]. Moreover, new research on empathy that incorporates developmental theory highlighted disturbances in prosocial behavior and sympathy (not empathy per se) may also be involved in the development of psychopathy [14]. Thus, understanding the development of psychopathy will likely need to involve more domains than CU traits (e.g., interpersonal, behavioral).

While CU-focused research emphasized genetic factors in understanding development of psychopathy, a more modern approach was recently offered, referred to as the ‘embedded brain’ [17]. The idea is that factors both external (e.g., family functioning, socioeconomic) and internal (e.g., neurocognitive development) to the child must be considered to understand the development of psychopathic personality. In other words, the “transactional and iterative unfolding of brain and cognitive development within a relational context” is perhaps the best approach for conceptualizing the development of psychopathic personality.

To study the emergence of psychopathic personality, being able to measure and compare psychopathic features across age validly and reliably, therefore, is of critical importance [18]. A structural model of psychopathic personality that generalizes across age would be optimal for studying the development of psychopathy, particularly one that involves traits that reflect relational factors (e.g., interpersonal and impulsive behavioral style), anti- versus pro-social propensities, and affective features.

### 1.1. The Four-Factor Model of Psychopathy

Among several existing conceptual models of psychopathy is the four-factor model, comprising interpersonal, affective, lifestyle, and antisocial facets. This model was adopted by many investigators [12] given robust statistical support [19] and validity with respect to age, race, gender, and cognitive ability in both community and clinical samples [2,3,20,21]. The interpersonal domain reflects features of grandiosity, glibness, pathological deception, and manipulation. The affective domain is described as a lack of remorse, lack of empathy, shallow affect, and failure to accept responsibility. The lifestyle domain represents impulsive, irresponsible, reckless, and sensation-seeking behaviors, whereas the antisocial domain is marked by early onset, pervasive, and versatile socially aversive behaviors. It is important to note that the antisocial factor is not equivalent to criminality per se [6], but rather a risk for violence and criminality [7,8].

The Psychopathy Checklist-Revised (PCL-R; [22])is one of the most widely used instruments for assessing psychopathic traits and behaviors in adults. Originally published in 1980, it measures socially aversive tendencies, aggressive behaviors, and deceitful interpersonal styles, alongside affective callousness and lack of remorse [22]. Its structure is based on the four-factor model of psychopathy, encompassing the Interpersonal, Affective, Lifestyle, and Antisocial Behavior factors [22]. Its strong empirical basis led to the creation of several derivative measures within the PCL family, such as the Psychopathy Checklist: Screening Version (PCL:SV; [23]) and Psychopathy Checklist: Youth Version (PCL:YV; [24]).

The PCL:YV assesses psychopathic traits and behaviors in adolescents ages 12 to 18 [24]. The youth-focused measure was adapted from and employs the same four-factor framework as its parent, the PCL-R [24]. Further, the PCL:YV is not intended for diagnosis or labeling youth as “psychopaths,” but rather for identifying precursors of psychopathy in development as well as points of intervention in the treatment program [25].

A clear methodological strength of the PCL-R and PCL:YV is that they involve semi-structured interviews employed by trained expert raters [22]. As is the case for all psychiatric assessments, PCL-R/YV interviews are considered standard assessment instruments, especially in forensic settings [26]. There are also self- and parent-report instruments that are based on the same four-factor model as the PCL instruments, and these instruments have robust associations with clinical correlates of psychopathy [2,19,27]. However, another methodological advantage of the PCL:YV is that behavior genetic research found that it reflects both genetic and environmental factors, while traditional self- or parent-report instruments appear to show potentially upwardly biased estimates of genetic effects for parent reports as well as non-specific genetic measurement effects [28]. Furthermore, to date, there are few self-report item sets that can be readily employed with both youths and adults, except perhaps the SRP-SF, which showed promise with adolescents and adults [19]. As such, expert PCL:YV (and PCL-R) ratings may be optimal for studying the development of psychopathy across age groups at this time.

Since the focus on developmental approaches in psychopathy research grew, important associations were identified between traits in adolescence and traits or outcomes in adulthood [18]. To start, increased psychopathic trait scores in youth were already linked to more severe and violent offenses and elevated risk of exhibiting psychopathic traits as adults [29]. Findings from follow-up studies indicate that higher PCL:YV scores are associated with greater risk for violence (10 years after assessment) [30] and convictions (up to 17 years after assessment) in adulthood [31]. PCL:YV factors also demonstrated predictive utility for outcomes in adulthood. For example, increased interpersonal behaviors in boys were associated with more psychopathic features [32] and antisocial tendencies with chronic re-offending [33] as an adult.

### 1.2. Measurement Invariance

An integral aspect of research on any psychological construct is the utility of assessing people of diverse backgrounds and at different timepoints. An assessment tool should measure a construct in the same manner across groups (i.e., without bias), which is established statistically through measurement invariance testing [20]. If an instrument works differently across groups, or lack of invariance is found, then issues regarding methodology (e.g., differences in administration) or measurement (e.g., different constructs being measured or changes in the construct of interest over time) may be present. Thus, without evidence of invariance, valid comparisons between groups will be suspect, and potentially, associations between the construct (e.g., psychopathy) and external correlates can be spurious. As a result, testing measurement invariance is critical in allowing for broader generalizations of findings [34].

While research on the invariance of psychopathic features across age is slower to emerge [2,18], research with the PCL-R provided relatively good evidence for its invariance across race/ethnicity [7,35], gender [34,36], and culture [27,37]. At the same time, previous measurement invariance and factor structure research on the PCL:YV itself is inconsistent and historically does not address invariance across ages. For instance, the PCL:YV was demonstrated to be invariant between White and Black American youths [38] and White and Indigenous Canadian youths [39], yet another study found noninvariance between White and Aboriginal youths [40]. Many studies validated the factor structure of the PCL:YV, with findings suggesting that both the three- and four-factor model provide best fit [41]. Additionally, the four-factor model has good fit and predictive utility for male justice-involved youths [42], as well as acceptable fit for female justice-involved youths [43]. Consequently, continuing this line of inquiry in invariance research, especially across age, is critical for supporting the construct validity of psychopathy.

Given the developmental nature of psychopathic personality, assessment of psychopathic features in youth is thought to reflect a “downward extension” of the adult construct, and therefore the potential application of a vast sum of adult psychopathy research to the youth construct [1]. While some argue that the downward extension lacks a developmental framework, the notion is supported by the established stability of psychopathic traits across age [18] and robust psychometrics of instruments such as the PCL-R/YV [44]. More specifically, research examining psychopathic traits in youth and adulthood established considerable similarities and consistencies, which suggest the measurement of a similar construct at the very least. For example, taxometric analyses of the construct indicate that, similar to the adult construct of psychopathy [45], psychopathic traits in youth are dimensional and exist on a continuum [46]. Using a general personality model and NEO scores, researchers found that psychopathic features in adolescence are stable over time and are not attributable to temporal inconsistencies (i.e., fluctuations in personality during teenage years) [47]. Psychopathic features in youth were also found to be stable into young adulthood using the Child Psychopathy Scale (CPS) and PCL:SV as well as the Minnesota Temperament Inventory [18,48]. Therefore, while the downward extension idea does not imply that youths can be diagnosed or labeled as psychopaths, the adult psychopathy literature can be useful in better understanding the developmental trajectory of the condition.

### 1.3. Current Study

The development of the PCL:YV is due, in part, to the vast literature on the PCL-R, and thus creates the opportunity to consider comparing PCL:YV and PCL-R scores among adolescents and adults. Of course, such a comparison may be difficult with most self-report instruments, except perhaps the Self-Report Psychopathy-Short Form scale (SRP-SF) [19]. Nevertheless, despite the conceptual and structural similarities between the PCL-R and PCL:YV, no prior studies examined whether they measure the construct of psychopathy consistently across age groups. Practically, the two instruments provide the opportunity to examine mean levels of the four psychopathic domains across developmentally different groups, and thus offer considerable opportunity to conduct future developmentally informed studies with these age groups. However, if the PCL-R and PCL:YV demonstrate non-invariance, the implications for false positives [49] within forensic and treatment settings are significant, as well as problematic comparisons. Consequently, our study aims to fill this gap in psychopathy assessment research to establish measurement invariance between the two measures to provide empirical support for valid and reliable comparisons across adolescents and adults.

A configural model (same structure but free loading and threshold parameters across groups), and strong (scalar) invariance model (equal item loadings and thresholds) were assessed to see if both PCL-R and PCL:YV models are equivalent across the adults and adolescents. If so, then latent means can be compared across groups meaningfully, which may shed light on the developmental expression of psychopathic features. In other words, establishing measurement invariance of the PCL-R and PCL:YV would suggest that any observed differences in levels of psychopathy trait domains (interpersonal, affective, lifestyle, and antisocial) may signify legitimate developmental differences rather than inconsistencies dues to measurement biases. Given the strong validity of the four-factor model of psychopathy across a variety of settings and samples, the following was hypothesized: (H1) The four-factor model structure will demonstrate good fit for adolescents and adults separately, as well as for a combined sample of adults/adolescents. (H2) Invariance of the four-factor model will be found across adolescents and adults. (H3) Latent means of the PCL factors will differ across samples, though the direction of such differences is an open area of investigation.

## 2. Methods

### 2.1. Study Design

The present study utilized quantitative expert ratings to determine measurement invariance of the four-factor model of psychopathy in a cross-sectional sample within a forensic setting. We used an exploratory structural equation modeling approach to model the factor structure of the PCL-R/YV and to test measurement invariance cross-sectionally.

### 2.2. Participants

Participants in the current study included *N* = 1091 incarcerated individuals (*n* = 278 adolescents and *n* = 813 adults) recruited from juvenile and adult correctional facilities in the state of New Mexico (USA). Participants ranged from 14.15 to 61.91 years of age (*M* = 30.40, *SD* = 10.70), with adults recruited from adult medium- to maximum-security correctional facilities ranging from 18.74 to 61.91 years of age (*M* = 34.70, *SD* = 8.97) and with adolescents recruited from a maximum-security juvenile correctional facility ranging from 14.15 to 19.89 years of age (*M* = 17.80, *SD* = 1.11). This age range is consistent with the New Mexico statute stating that youths may be committed to the care of the juvenile department until the age of 21, in accordance with Section 32A-2-23 NMSA 2023 [50]. The age range is also consistent with definitions by the World Health Organization, which considers the developmental period of adolescence to range from 10 to 19 years of age.

Regarding NIH race and ethnicity classifications, participants self-identified as either American Indian or Alaskan Native (adolescents: *n* = 31; adults: *n* = 68), Asian (adolescents: *n* = 0; adults: *n* = 5), Black or African American (adolescents: *n* = 15; adults: *n* = 76), Native Hawaiian or other Pacific Islander (adolescents: *n* = 1; adults: *n* = 0), White (adolescents: *n* = 176; adults: *n* = 634), or more than one race (adolescents: *n* = 10; adults: *n* = 11); additionally, *n* = 45 adolescents and *n* = 19 adults chose not to self-disclose their race. Regarding ethnicity, participants self-identified as Hispanic or Latino (adolescents: *n* = 209; adults: *n* = 472) or not Hispanic or Latino (adolescents: *n* = 65; adults: *n* = 341); additionally, *n* = 4 adolescents chose not to self-disclose their race. Table 1 displays the demographic characteristics of the total sample.

### 2.3. Procedures

Initial contact was made with study participants by research staff from the Mind Research Network and informed consent was obtained. Specifically, individuals 18 years of age or older provided written informed consent and individuals younger than 18 years of age provided written informed assent in conjunction with a parent or legal guardian’s informed consent. Interested study participants were excluded from participating in our research studies if they were (1) missing criminal or institutional files to supplement the semi-structured interview or (2) characterized by deficiencies that would impact their ability to properly consent to the overall research study or accurately complete the PCL-R/PCL:YV. For example, participants included in the current study were not characterized by a major medical condition (e.g., epilepsy, cancer, severe traumatic brain injury, brain tumor, etc.) and had a full-scale intelligence quotient (IQ) score above 70 and at least a fourth-grade reading level. Participants received payment consistent with the hourly labor wage of the correctional facility they were recruited from. All research protocols were approved by the University of New Mexico Human Research Review Committee and the Office for Human Research Protections.

### 2.4. Measures

Psychopathic traits were assessed among adolescents via the PCL:YV [24] and among adults with the PCL-R [22]. Both the PCL:YV and PCL-R are expert-administered rating scales, consisting of a semi-structured interview and review of collateral information, including institutional files. Items of the PCL:YV and PCL-R overlap significantly with one another, with items being modified for age appropriateness for the PCL:YV (e.g., the item “promiscuous sexual behavior” in the PCL-R is modified to “impersonal sexual behavior” in the PCL:YV). Each of the twenty items of the PCL:YV and PCL-R are rated on a three-point scale (0, 1, and 2), with scores of zero indicating the item does not apply to the individual, scores of one meaning the item applies somewhat to the individual, and scores of two indicating the item definitely applies to the individual, with PCL:YV and PCL-R total scores ranging from zero to 40.

Administration and ratings of the PCL:YV and PCL-R were completed by trained research staff employed by the senior author, all of whom had a bachelor’s degree or higher education level. All research staff who administered the PCL:YV and PCL-R completed a rigorous training process designed and supervised by Dr. Kent Kiehl, who was directly trained by Dr. Robert Hare. Previous research from the New Mexico lab demonstrated good inter-rater reliability [51].

Based on the semi-structured interview and review of collateral information (e.g., institutional files and criminal record review), the majority of the twenty items included in the PCL-R and PCL:YV were able to be sufficiently scored for each participant. For any items that were omitted based on insufficient details, prorated PCL-R and PCL:YV scores were calculated [22]. For adolescents, *n* = 9 participants had one item omitted, *n* = 4 participants had two items omitted, and *n* = 1 participant had three items omitted. For adults, *n* = 274 participants had one item omitted, *n* = 57 had two items omitted, *n* = 43 participants had three items omitted, *n* = 38 participants had four items omitted, and *n* = 9 participants had five items omitted.

### 2.5. Preliminary Analyses

For adolescents, the mean PCL:YV total score was 23.48 (*SD* = 6.07, range: 2–35, and α = 0.83) and for adults, the mean PCL-R total score was 20.60 (*SD* = 6.75, range: 3.2–38, and α = 0.81). In addition to PCL:YV and PCL-R total scores, we also investigated factor and facet structures from these instruments. Both the PCL:YV and PCL-R show a similar two-factor and four-facet structure, with Factor 1 measuring interpersonal psychopathic traits (i.e., Facet 1 items including pathological lying and a grandiose sense of self-worth), and affective psychopathic traits (i.e., Facet 2 items, such as a lack of remorse or guilt and shallow affect) and Factor 2 assesses lifestyle/behavioral psychopathic traits (i.e., Facet 3 traits, including stimulation seeking and impulsivity), and antisocial/developmental psychopathic traits (i.e., Facet 4 traits, such as criminal versatility and poor anger/behavioral controls) [1,22,24,52]. For adolescents, the mean PCL:YV Factor 1 score was 6.65 (*SD* = 3.07, range: 0–15, and α = 0.73) and the mean PCL:YV Factor 2 score was 14.57 (*SD* = 3.24, range: 1–20, and α = 0.73). For adults, the mean PCL-R Factor 1 score was 5.69 (*SD* = 3.72, range: 0–15, and α = 0.74) and the mean PCL-R Factor 2 score was 12.70 (*SD* = 3.72, range: 1.1–20, and α = 0.71).

For adolescents, the mean PCL:YV Facet 1 score was 2.19 (*SD* = 1.85, range: 0–7, and α = 0.67, MIC = 0.34), the mean PCL:YV Facet 2 score was 4.46 (*SD* = 1.79, range: 0–8, α = 0.60, and MIC = 0.28), the mean PCL:YV Facet 3 score was 6.30 (*SD* = 2.01, range: 0–10, α = 0.62, and MIC = 0.25), and the mean PCL:YV Facet 4 score was 8.29 (*SD* = 1.65, range: 0–10, α = 0.56, and MIC = 0.25). For adults, the mean PCL-R Facet 1 score was 2.04 (*SD* = 1.92, range: 0–8, α = 0.69, and MIC = 0.37), the mean PCL-R Facet 2 score was 3.65 (*SD* = 2.02, range: 0–8, α = 0.63, and MIC = 0.30), the mean PCL-R Facet 3 score was 5.52 (*SD* = 2.15, range: 0–10, α = 0.58, and MIC = 0.23), and the mean PCL-R Facet 4 score was 7.20 (*SD* = 2.26, range: 0–10, α = 0.64, and MIC = 0.27). Mean inter-item correlations (MICs) for each facet were acceptable and preferred, given that Cronbach’s alpha is influenced by scale length and not a precise measure of scale homogeneity [53]. The observed (manifest variable) PCL-R/YV score differences for the adult versus adolescent samples are displayed in Table 2.

### 2.6. Primary Analyses

Exploratory structural equation modeling (ESEM) is a statistical analysis technique that allows for items to cross-load on multiple factors. This approach was utilized because it has the advantages associated with confirmatory factor analysis (CFA; goodness-of-fit) without its restrictions (e.g., items only loading onto main factors and crossloadings set to 0). As such, ESEM became a preferred approach when modeling personality data [54]. As an added benefit, ESEM decreases the likelihood of inflated factor correlations given it accounts for item cross-loadings and therefore can help provide more differentiated and precise estimates of factor associations. Thus, we conducted ESEM to examine model fit of the four-factor PCL-R/YV structure. Next, using multiple-group ESEM (MG-ESEM), measurement invariance of the PCL-based four factor model was tested across adolescent and adult samples by comparing the fit of the configural model to the scalar model.

The analyses were conducted in Mplus 8.4 [55] using weighted least squares mean and variance-adjusted (WLSMV) estimation, given the ordinal PCL-R/YV items. To test an ESEM of the four-factor model, the PCL-R/YV items were allowed to freely load onto each of the four factors, and factors were allowed to correlate. The following goodness-of-fit indices were utilized: the comparative fit index (CFI), the Tucker–Lewis index (TLI), the root mean square error of approximation (RMSEA), and the standardized root mean square residual (SRMR). Since model complexity can impact conventional fit [56], adequate and excellent standards of CFI (≥0.90 and ≥0.96), TLI (≥0.90 and ≥0.95), RMSEA (≤0.08 and ≤0.05), and SRMR (≤0.08) were used as benchmarks for model fit to avoid false rejections of potential latent variable models [57]. Lastly, because the chi-square (χ^2^) test of exact fit is sensitive to sample size and cannot be used with WLSMV for chi-square difference testing, it carried less emphasis but was nevertheless reported.

To compare configural and scalar models, we examined changes (Δ) in the goodness-of-fit indices, with evidence for invariance if ΔCFI was ≤0.010 or ΔRMSEA was ≤0.015 [56,58]. To be comprehensive, we also present ΔTLI and ΔSRMR. Notably, suggested changes in CFI and RMSEA for assessing invariance are based on CFA, and therefore, we followed recent guidelines for studies that employ multiple-group ESEM [59]. Additionally, given that assessing change in fit can be more challenging when using ordinal data [60], we also used maximum likelihood estimation (treating the items as continuous), given our relatively large overall sample size and that the sample size adjusted Baysian information criteria (BIC_adj_) also provides information regarding scalar invariance [59].

## 3. Results

Good fit for a four-factor ESEM model was demonstrated in both the combined and individual samples. The goodness-of-fit indices for this model are displayed in Table 3.

**Figure 1:**
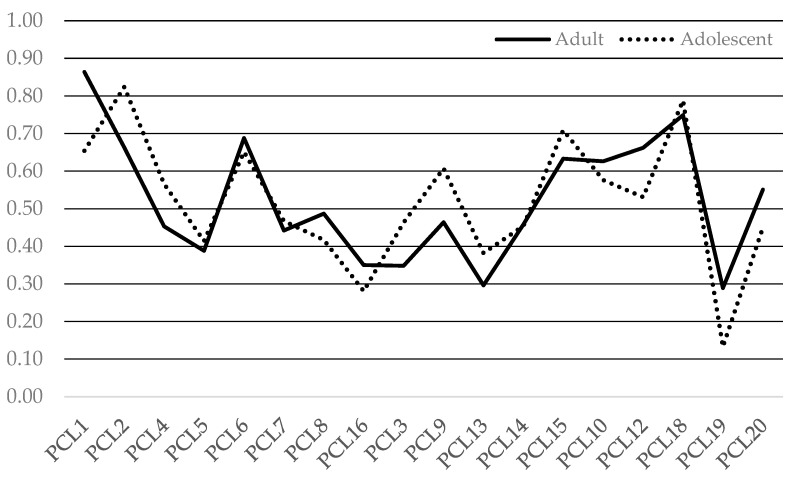
Standardized discrimination (factor loading) parameters by sample (scalar model).

**Figure 2:**
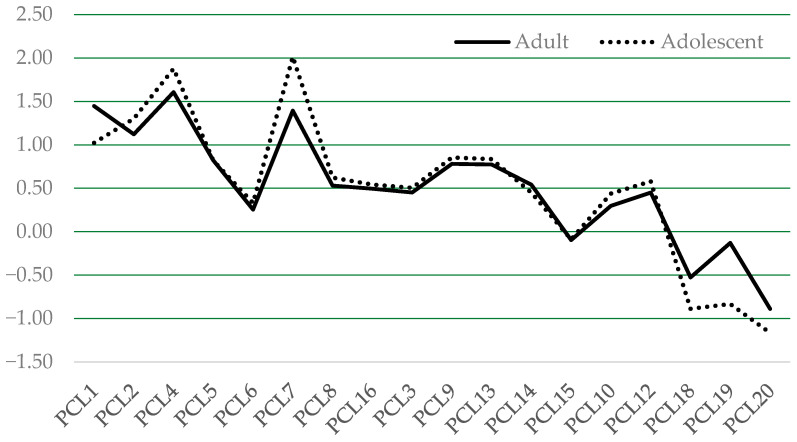
Threshold (b_2_) parameters by sample (scalar model). Note: Here, b_2_ thresholds depict the degree of latent psychopathic propensity required to increase the likelihood of meeting a trait rating of 2 (present) on a given item. Generally concordant thresholds indicate that the item tap constructs severity similarly across groups, providing evidence of measurement invariance.

The results from the MG-ESEM provide generally good evidence for strong (scalar) invariance (equivalent loadings and thresholds). Table 4 presents the fit statistics for the MG-ESEM that tested configural and scalar invariance of the PCL-R/YV items across groups. The standardized parameter estimates (also known as factor loadings) for the adolescent and adult samples are presented in Table 5 and Table 6; they are also presented together visually in Figure 1. The threshold parameters are visually displayed in Figure 2.

The incremental fit difference (ΔCFI) was slightly above the 0.010 standard for invariance, though the ΔTLI was below 0.010. The absolute fit index difference (ΔRMSEA) met the 0.015 standard for invariance. Examination of the pattern of item thresholds from the configural model suggested that freeing the b_2_ thresholds (i.e., ratings of 2) for items 9 and 18 could slightly improve fit (see Scalar * results in Table 4). Additionally, it is reasonable to suggest that these specific items (parasitic lifestyle, juvenile delinquency) may be rated slightly differently in adolescents versus adults (i.e., different thresholds) due to differences in developmental stage. As it turned out, freeing these threshold parameters did increase the evidence for (partial) scalar invariance (ΔCFI = 0.014). Finally, when the same multiple group ESEM was run via maximum likelihood estimation, the BIC_adj_ index provided additional support for good fit for the scalar model via a smaller BIC_adj_ (35,671), compared to the configural model (BIC_adj_ = 35,698). Taken together, acceptable evidence of strong invariance was found across the adults and adolescents.

The PCL item-to-factor loadings demonstrated that items loaded primarily on their designated factor with generally minimal cross-loadings. However, there were several notable cross-loadings, such as item 4 on the lifestyle factor, item 8 on the antisocial factor, and items 19 and 20 on the lifestyle factor. This pattern of item cross-loadings were similar for the adults and adolescents.

As can be seen in Figure 1 and Figure 2, there was considerable uniformity of the item discrimination (loadings) and threshold parameters across the adults and adolescents, indicating that the PCL-R/YV items are able to similarly identify adults and adolescents who vary in psychopathic features, and at the same level of psychopathic severity.

While the MG-ESEM scalar model constrains loadings and thresholds to be similar across adults and adolescents, this model allows factors correlations to be freely estimated across the two groups. Notably, for the adults, there were stronger correlations between the interpersonal factor with the affective (*r* = 0.39), lifestyle (*r* = 0.32), and antisocial (*r* = 0.24) factors, compared to the adolescents, affective (*r* = 0.14), lifestyle (*r* = 0.08), and antisocial (*r* = 0.21). Conversely, for the adolescents, the affective factor was more strongly correlated with the lifestyle (*r* = 0.37) and antisocial (*r* = 0.63) factors, compared to the adults, lifestyle (*r* = 0.21), and antisocial (*r* = 0.10). Additionally, the association between the lifestyle and antisocial factor for adolescents (*r* = 0.89) was about twice that as for adults (*r* = 0.46).

**Figure 3:**
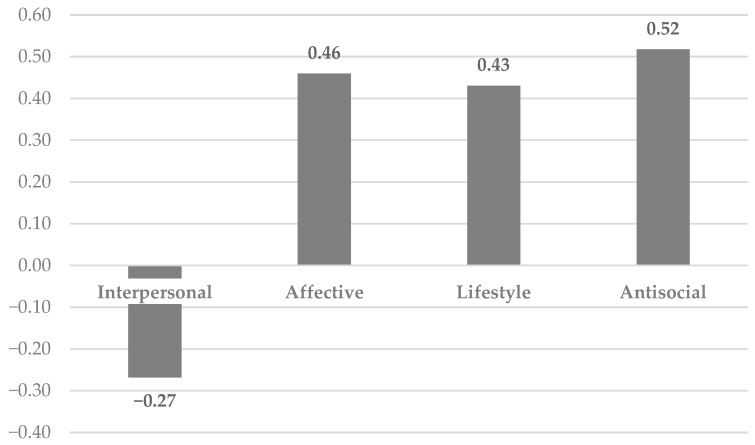
Adolescent PCL latent means relative to those of adults. Note: Adult PCL latent means were set at 0 by default in *Mplus* to provide group comparisons.

Lastly, the MG-ESEM results indicate that there were significant differences in the latent means between the adults versus adolescent samples. Specifically, adolescents displayed statistically significantly lower latent interpersonal traits than adults, but higher latent features on the other PCL factors. These findings are displayed in Figure 3.

## 4. Discussion

The results provide continued support for the four-factor model of psychopathy [1] with the PCL-R/YV items loading on their respective factor. There were some notable item cross loadings, though this may be expected given the likely covariation of Factor 1 and Factor 2 features among individuals with psychopathic personality tendencies (e.g., affective callousing that covaries with aggressive antisocial behavior). Evidence for such covariation was documented in person-centered research among subtypes of adults [61] adolescents [62] with latent prototypic psychopathy profiles. Moreover, the ESEM approach was useful for uncovering the same four psychopathy factors (interpersonal, affective, lifestyle, and antisocial) among justice-involved adults and adolescents.

The evidence for measurement invariance of the PCL-R/YV items indicate that conceptually and empirically, the four-factor model remains valid across the adult and adolescent samples. More specifically, the respective item sets worked similarly in discriminating adults and adolescents who vary in psychopathic propensities and at the same level of the underlying latent trait. Thus, the PCL:YV can be considered a “downward extension” of the PCL-R to the extent that the same latent factors were statistically represented for adults and adolescents. Nevertheless, while our results provide support for a downward extension, they still do not provide any reason for diagnosing youths as psychopaths. Practically, what this translates to is that there is robust support for valid mean PCL-R/YV comparisons among adults vs. adolescents with psychopathic features. Thus, the findings from our study should assist in future research that seeks to examine what developmental biopsychosocial factors can account for latent PCL-R/YV mean scores.

The notable differences in the latent mean scores are intriguing, though their exact implications currently are unclear. Our results showing lower latent interpersonal traits among adolescents compared to adults suggest that interpersonal psychopathic features may increase with development. This interpretation can be contextualized with respect to behavior genetic research on the PCL:YV, which found that environmental effects accounted for interpersonal traits among male adolescents [28]. Thus, on-going relational experiences may shape the emergence of interpersonal psychopathic style over time in line with the embedded brain perspective —i.e., a “dynamic interplay between individuals and their social ecology” [17] (p. 159). In addition, our finding of stronger correlations between the interpersonal factor and the other psychopathy factors among the adults, compared to adolescents, supports this line of thinking. Put another way, if interpersonal traits develop more slowly over time than other psychopathic features which emerge earlier (e.g., antisocial and CU traits; [63]), then it is reasonable to expect that interpersonal traits should be more integrated (i.e., covary more strongly) with other psychopathic propensities among adults, compared to adolescents. On the other hand, evidence of lower latent means of the lifestyle and overt antisociality domains among adults is consistent with the idea of the ‘burnout’ of Factor 2 traits with age across both men and women [64,65]. Finally, our results showing high levels of affective traits, as well as Factor 2 traits, among the adolescents, compared to the adults, along with stronger affective factor associations with the other psychopathy factors, are consistent with evidence that overt antisociality and affective callousness are both core and early emerging covarying aspects of psychopathic personality [2,6,19]. In particular, the behavior genetic findings reported by Viding and colleagues [63] that affective and overt behavioral antisocial features reflect a common genetic factor helps to account for our pattern of correlations. Conversely, for adults, the so-called ‘burnout’ of Factor 2 traits and the plethora of reasons why adults may engage in antisocial behavior (e.g., establishment of criminal lifestyle) helps to interpret a weaker pattern of psychopathy factor associations aside from psychopathic propensity.

In sum, the findings provide continued support for the notion that psychopathy is a lifelong condition, and the early manifestations of psychopathic personality can be traced back to adolescence. Therefore, our findings that the PCL-YV and PCL-R reflect a similar latent construct across age reinforces the emphasis on early identification and intensive intervention [66]. At the same time, our results suggest that there may be changes in the expression in the levels of specific psychopathic features with development. As such, our results highlight the need for longitudinal research, including studies that examine both adults and adolescents, to help uncover how changes in development (particularly neurocognition) may be linked to the social ecology in which people live [17].

The implications of these findings are not only important for researchers and clinicians, but also anyone involved in forensic decision making. It is critical to highlight that even with consideration of our findings, PCL:YV scores should not be utilized to preclude an individual from treatment, lengthen sentencing, or sway probation decisions. PCL:YV scores, at their core, should be utilized for short-term clinical decision making (e.g., determining treatment needs, evaluating symptom severity) only. Forensic dispositions may also benefit from the inclusion of PCL:YV scores in determining type of placement for youth to best suit their clinical needs. In research settings, tracking psychopathic traits and how they manifest throughout the lifespan can help identify protective/risk factors and changes in symptom trajectory, thereby informing rehabilitation efforts and treatment efficacy studies.

Of course, this study was not without its limitations. Firstly, the quality and consistency of PCL-R/YV training can impact item ratings, especially considering the influence of rater–participant interactions. Thus, a strength of our study involved highly trained expert raters who were well-versed in the administration of the state-of-the-art semi-structured PCL-R/YV interviews. Participant gender was restricted due to institutional limitations (e.g., male-only facilities). Therefore, forthcoming research should focus on measurement invariance across female adolescents and adults from their respective institutions. However, another strength of our study is that we used samples where the prevalence of elevated levels of psychopathic propensity was assured. At the same time, while the racial and ethnic breakdown of the sample resembles the demographics of New Mexico, where the data were collected [67], it is noted that these percentages are less representative of the United States as a whole [68]. Still, efforts in ensuring sample diversity should be continued to find support for cross-gender, cross-cultural, and cross-cohort measurement invariance. Finally, future measurement invariance studies involving the PCL-R and PCL:YV would also undoubtedly benefit from examining invariance longitudinally (i.e., PCL:YV and PCL-R scores from the same individual are compared).

## Figures and Tables

**Table 1:** Total Sample Demographics (*N* = 1091).

Variable	Group	n	%
Cohort	Adolescent	278	25.48
	Adult	813	74.52
Race	American Indian/Alaska Native	99	9.07
	Asian	5	0.46
	Black/African American	91	8.34
	Native Hawaiian/Other Pacific Islander	1	0.09
	White	810	74.24
	More than one	21	1.92
	Unknown/Not reported	64	5.87
Ethnicity	Hispanic/Latino	681	62.42
	Not Hispanic/Latino	406	37.21
	Unknown/Not reported	4	0.37

**Table 2:** Descriptive Statistics for PCL:YV and PCL-R Scores.

	Adolescents (n = 278)		Adults (n = 813)		Group Differences		
Variable	Mean	Std. Dev.	Mean	Std. Dev.	*T*	df	*p*-Value
PCL Total	23.48	6.07	20.60	6.75	6.29	1089	<0.001
PCL Factor 1	6.65	3.07	5.69	3.35	4.22	1089	<0.001
PCL Factor 2	14.57	3.24	12.70	3.72	7.49	1089	<0.001
PCL Facet 1	2.19	1.85	2.04	1.92	1.15	1089	0.252
PCL Facet 2	4.46	1.79	3.65	2.02	5.90	1089	<0.001
PCL Facet 3	6.30	2.01	5.52	2.15	5.31	1088	<0.001
PCL Facet 4	8.29	1.65	7.20	2.26	7.35	1071	<0.001

Note. Factor 1 = interpersonal-affective traits; Factor 2 = lifestyle-antisocial traits; Facet 1 = interpersonal traits; Facet 2 = affective traits; Facet 3 = lifestyle traits; and Facet 4 = overt antisocial traits.

**Table 3:** Fit Statistics of Four-Factor ESEM Model: Total, Adult, and Adolescent Samples.

	χ^2^	*df*	RMSEA (90% CI)	CFI	SRMR	TLI
Total sample	311.603	87	0.049 (0.043–0.055)	0.965	0.036	0.938
Adolescent	126.747	87	0.041 (0.024–0.055)	0.976	0.054	0.958
Adult	264.404	87	0.050 (0.043–0.057)	0.961	0.039	0.931

**Table 4:** Fit Statistics for MG-ESEM: Adults/Adolescents.

	χ^2^	*df*	RMSEA (90% CI)	ΔRMSEA	CFI	ΔCFI	SRMR	ΔSRMR	TLI	ΔTLI
Configural	371.450	174	0.046 (0.039–0.052)		0.967		0.043		0.942	
Scalar	562.502	244	0.049 (0.044–0.054)	0.003	0.947	0.020	0.059	0.016	0.934	0.008
Scalar *	526.172	242	0.046 (0.041–0.052)	0.000	0.953	0.014	0.059	0.016	0.940	0.002

Note. “*” = Partial scalar invariance (i.e., free b_2_ thresholds for items 9 and 18).

**Table 5:** Adolescent PCL Factor Loadings.

PCL:YV Items	INT	AFF	LIF	ANT
Interpersonal				
1	0.65	0.04	0.04	0.01
2	0.82	0.18	−0.05	0.04
4	0.57	0.03	0.69	−0.16
5	0.41	−0.06	0.45	0.06
Affective				
6	0.01	0.65	0.01	0.25
7	0.15	0.47	0.21	0.05
8	0.04	0.42	0.02	0.47
16	0.30	0.28	0.01	−0.05
Lifestyle				
3	0.21	−0.05	0.46	0.16
9	0.15	0.00	0.61	0.02
13	0.06	0.30	0.38	−0.06
14	−0.13	0.01	0.45	0.15
15	−0.13	0.04	0.71	0.00
Antisocial				
10	0.02	0.14	−0.06	0.58
12	0.07	0.05	0.01	0.53
18	−0.14	−0.02	0.07	0.79
19	−0.16	0.04	0.35	0.13
20	0.05	−0.28	0.71	0.45

Note: All factor loadings significant (*p* < 0.05–0.0001) except those less than 0.10.

**Table 6:** Adult PCL Item Factor Loadings.

PCL-R Items	INT	AFF	LIF	ANT
Interpersonal				
1	0.86	0.07	0.04	0.02
2	0.66	0.21	−0.04	0.06
4	0.45	0.04	0.50	−0.22
5	0.39	−0.08	0.38	0.10
Affective				
6	0.01	0.69	0.00	0.31
7	0.10	0.44	0.12	0.05
8	0.03	0.49	0.02	0.64
16	0.26	0.35	0.00	−0.07
Lifestyle				
3	0.18	−0.07	0.35	0.23
9	0.13	−0.01	0.46	0.04
13	0.05	0.38	0.30	−0.09
14	−0.15	0.02	0.46	0.30
15	−0.13	0.05	0.63	0.00
Antisocial				
10	0.01	0.13	−0.03	0.63
12	0.05	0.05	0.01	0.66
18	−0.08	−0.02	0.03	0.75
19	−0.20	0.08	0.40	0.29
20	0.04	−0.29	0.45	0.55

Note: All factor loadings significant (*p* < 0.05–0.0001) except those less than 0.10.

## Data Availability

Dataset available on request from the authors.
